# Fracture of the Inferior Maxilla

**Published:** 1858-07

**Authors:** Charles Knower

**Affiliations:** St. Louis.


### ARTICLE XII.

*Fracture of the Inferior Maxilla.* A case reported by Mr. Charles Knower, of St. Louis.

John Monoghan came to the "St. Louis Dispensary" on the 20th of March, seeking aid for internal injuries, and also to have the left anterior inferior bicuspid extracted, which had been troublesome since the 6th instant, on which day a pair of horses attached to a heavy loaded wagon ran away, throwing him with violence to the ground, and the wagon passing over him, from which time he had not been able to articulate the front teeth, within nine lines of their normal condition, and entirely unable to masticate solid food, but compelled to subsist on fluids;—he did not feel alarmed, thinking the loose tooth was the only cause of trouble, and by having it removed, the trouble would cease. After receiving the history of the case, and on making an examination, I found a space some six lines in width between the anterior and posterior bicuspids, but by applying some force to the anterior bicuspid, I was enabled to bring it quite to its natural position, and at the same time I detected that the front part of the jaw was elevated and moved back with it, which I knew could not be a normal condition; and I immediately proceeded to ferret out the cause, the result of which was the diagnosis of a fracture of the left body, commencing at a point on the base some six lines from the angle, running obliquely in a line with the jaw, to the point indicated between the bicuspids. Having learned the nature of the case I had to deal with, I adjusted the Gibson or paste-board splint, moistened in warm water before applying to the chin, placing suitable compresses underneath, and secured with the four-tailed bandage; at the first application I was not enabled to bring the teeth in direct articulation as was natural, which was owing to the *callus* that had been formed in the interval from the time of accident to time of receiving patient—a period of two weeks; directed the patient to keep the jaw quiet and return daily and have the application renewed, by which means the *callus* would be displaced, and the jaw brought to its normal condition.

March 22d.—By tightening the bandage I found some improvement.

23d.—Tightened again with improvement.

24th.—The patient returned with bandage nearly off, and jaw in as bad condition as when first seen. I inquired as to how the bandage became loosened, and his response was, that the wife thinking it *too* dirty had removed for the purpose of washing, regardless of the trouble and danger she was creating, and had again adjusted to the best of her ability. I again made it secure, and gave positive orders not to have the bandage removed again under any circumstances.

25th.—Patient returned, having a very violent cough, which I apprehend will delay progress. I tightened the bandage, and to my gratification, as well as the patient, found the teeth articulate naturally.

April 3d.—There has been no change.

April 10th.—Three weeks have passed since first application of bandage; the jaw remains in its normal condition, and every thing favorable to a speedy and good result.

CHAS. KNOWER.

---

[At our solicitation the preceding report was prepared by Mr. Knower. We urged it for the development of *one fact* we would fix in the memory of every student of dentistry. Mr. Knower is a young man, and has been a student (of Dr. Isaiah Forbes) but one year. Has attended but one course of lectures, (just closed at the St. Louis Medical College;) but yet by the *experience* thus gained, he was enabled at the outset to avoid an evil which older, but less patient, operators might easily have fallen into—the extraction of the indicated tooth. As Mr. K. is studying dentistry, ailments of the teeth presenting at the infirmary fall into his hands, and this case was handed over to him that he might extract the offending member. Fortunately for the reputation of dentistry, he had been taught to examine before extraction, and thus was the true evil discovered and remedied, and credit gained by himself and his instructors. To the young who are aspiring to be dentists, we say, if you wish for success, be *students* first. The rest is easy.—ED. *Am. Dental Review.*]

